# Predictive performance of the mHAP-II score in a real-life western cohort with hepatocellular carcinoma following trans-arterial chemoembolisation with drug-eluting beads (DEB-TACE)

**DOI:** 10.1007/s00330-020-06734-8

**Published:** 2020-03-03

**Authors:** Felix Peisen, Michael Maurer, Ulrich Grosse, Konstantin Nikolaou, Roland Syha, Dominik Ketelsen, Christoph Artzner, Michael Bitzer, Marius Horger, Gerd Grözinger

**Affiliations:** 1 Department of Diagnostic and Interventional Radiology, Eberhard Karls University, Hoppe-Seyler-Strasse 3, 72076 Tübingen, Germany; 2 Department of Radiology, Kantonsspital Münsterlingen, Spitalcampus 1, 8596 Münsterlingen, Switzerland; 3 Department of Diagnostic and Interventional Radiology, Prosper Hospital Recklinghausen, Mühlenstrasse 27, 45659 Recklinghausen, Germany; 4 Ihre-Radiologen.de MVZ GmbH, Interventional and Diagnostic Imaging Centers, Heinz-Galinski-Strasse 1, 13347 Berlin, Germany; 5 Department of Gastroenterology, Gastrointestinal Oncology, Hepatology and Infectious Diseases, Eberhard Karls University, Otfried-Müller-Str. 10, 72076 Tübingen, Germany

**Keywords:** Carcinoma, hepatocellular, Chemoembolisation, therapeutic, Microspheres, Prognosis, Retrospective studies

## Abstract

**Objectives:**

To evaluate the predictive performance of the modified hepatoma arterial embolisation prognostic II (mHAP-II) score in a real-life western hepatocellular carcinoma (HCC) cohort treated with drug-eluting bead-TACE and compare the mHAP-II with other scores in this cohort.

**Methods:**

One hundred seventy-nine HCC patients (mean age 77 (± 9) years, 87% male) with one or more drug-eluting bead (DEB)-TACE sessions using 100–300 μm microspheres were retrospectively analysed. Performance analysis of the mHAP-II score was based on Mann-Whitney *U* tests, the Kaplan-Meier method, log-rank tests, receiver operating characteristics, Akaike’s information criterion and Cox regression models.

**Results:**

In this population, HCC risk factors were mainly alcohol abuse (31%) and hepatitis C (28%). The median survival of the entire cohort was 29.4 months. mHAP-II classification of the cohort was mHAP-II B (30%), C (41%) and D (23%) respectively. Survival of all subgroups differed significantly from each other (each *p* < 0.05). Area under the curve for receiver operating characteristic was 0.60 and Akaike’s information criterion was 21.8 (*p =* 0.03), indicating a superior performance of mHAP-II score compared with HAP score and BCLC. Tumour number ≥ two (HR 1.54), alpha-fetoprotein > 400 μg/l (HR 1.14), serum albumin < 3.6 g/dl (HR 1.63) and total bilirubin > 0.9 mg/dl (HR 1.58) contributed significantly in Cox proportional hazards regression (each *p* < 0.05).

**Conclusion:**

The mHAP-II score can predict survival outcomes of western HCC patients undergoing DEB-TACE and further subdivide this heterogeneous group; however, certain limitations concerning the predictive power of mHAP-II score must be taken into account.

**Key Points:**

*• This retrospective study evaluated the predictive performance of the modified hepatoma arterial embolisation prognostic II (mHAP-II) score in a real-life western HCC cohort treated with drug-eluting bead-TACE.*

*• Survival of all mHAP-II subgroups differed significantly, area under the curve for mHAP-II was 0.60 and Akaike’s information criterion was 21.8.*

*• The mHAP-II score can predict survival outcomes of western HCC patients undergoing DEB-TACE and further subdivide this heterogeneous group. However, because the study is underpowered, true survival prediction may be more difficult to infer.*

## Introduction

Patients with Barcelona Clinic Liver Cancer (BCLC) stage B hepatocellular carcinoma (HCC) have multinodular tumours, mildly or moderately impaired liver function (Child-Pugh A and B) and present with no or mild clinical disease symptoms (Eastern Cooperative Oncology Group (ECOG) performance status (PS) 0). Current guidelines suggest that palliative treatment with trans-arterial chemoembolisation (TACE) is the preferred treatment method for this patient group. [1]. Patients with BCLC stage 0 or A who are candidates for liver transplantation (LTX) can also receive TACE as a bridging therapy [2, 3]. The BCLC-B cohort, however, is very heterogeneous with regard to tumour burden and liver function [4]. As such, the effectiveness and benefit of TACE varies greatly amongst BCLC-B patients. Some attempts have been made to further subdivide the BCLC-B cohort to better predict the benefits of TACE treatment by using different scoring systems such as the OKUDA score [5], the CLIP score [6], the MESH score [7], as well as the SNACOR risk prediction model [8]. One of the most recent scoring systems is the so-called hepatoma arterial prognostic score (HAP score), introduced in 2013 by Kadalayil et al [9]. This score includes parameters for liver function (1 point each for albumin < 36 g/dl or bilirubin > 17 μmol/l) and tumour burden (1 point each for alpha-fetoprotein (AFP) > 400 ng/ml and tumour size > 7 cm). Patients can then be divided into four subgroups according to their score (HAP A = 0 to HAP D ≥ 3 points). Pinato et al presented a modified version in 2015, the so-called mHAP score [10]. Here, the parameter bilirubin was excluded, and the criterion of portal vein involvement was added in combination with the post-treatment variable “modified response evaluation criteria in solid tumours” (mRECIST). A third version, the mHAP-II score, was introduced by Park et al in 2015 [11]. In accordance with the HAP score, it contained the parameters albumin (< 36 g/dl) and bilirubin (> 17 μmol/l) for liver function, AFP (> 400 ng/dl) and tumour size (> 7 cm) for tumour burden and additionally rated the lesion number (≥ two).

Park et al showed an improved prognostic performance compared with HAP score and mHAP score both in a static risk assessment (categorisation before first TACE) and a dynamic risk assessment (sequential re-categorisation after first TACE) [11, 12]. Their Korean study population showed the typical aetiologic pattern of Asian populations, with hepatitis B virus (HBV) infection being the main risk factor for HCC (70%). However, in a western population, hepatitis C virus (HCV) infection and alcohol abuse are the predominant risk factors (60–70% and 20%, respectively) [13–15]. Alcohol might play an even more dominant role in northern Europe, with about one-third of HCC patients suffering from liver cirrhosis due to alcohol abuse [16, 17].

A new technique for TACE has been introduced over the last decade [18], the so-called drug-eluting bead-TACE (DEB-TACE). DEB-TACE combines local chemotherapy and embolisation by preloading the microspheres with a chemotherapeutic agent (e.g. doxorubicin or epirubicin) [18, 19].

Because of the fundamental differences in (disease) aetiology from which the scoring systems evolved, the question arises as to whether the mHAP-II score is still suitable for a further subdivision of BLCL-B HCC patients, and how its performance compares to other scoring systems in a western population treated with DEB-TACE.

We therefore evaluated the predictive performance of the mHAP-II score in a real-life western HCC cohort of 179 patients treated with DEB-TACE and compared it to other known scoring systems (HAP, OKUDA, CLIP, MESH and BCLC).

## Material and methods

### Study population

Two hundred twenty-eight patients with HCC undergoing their first DEB-TACE between June 2006 and March 2016 were retrospectively identified from a database in our department. Exclusion criteria were DEB-TACE of tumours other than HCC, Child-Pugh class C, extra-hepatic disease, central portal vein thrombosis, hepatic encephalopathy and/or refractory ascites.

HCC was diagnosed histologically and/or via imaging tools (contrast-enhanced MRI or multiphase contrast-enhanced CT) according to current EASL (European Association for the Study of the Liver) guidelines [20]. Criteria for a positive finding of HCC on dynamic CT or MRI were increased arterial enhancement of the lesion compared with the portal or equilibrium phase (so-called washout).

**Fig. 1:**
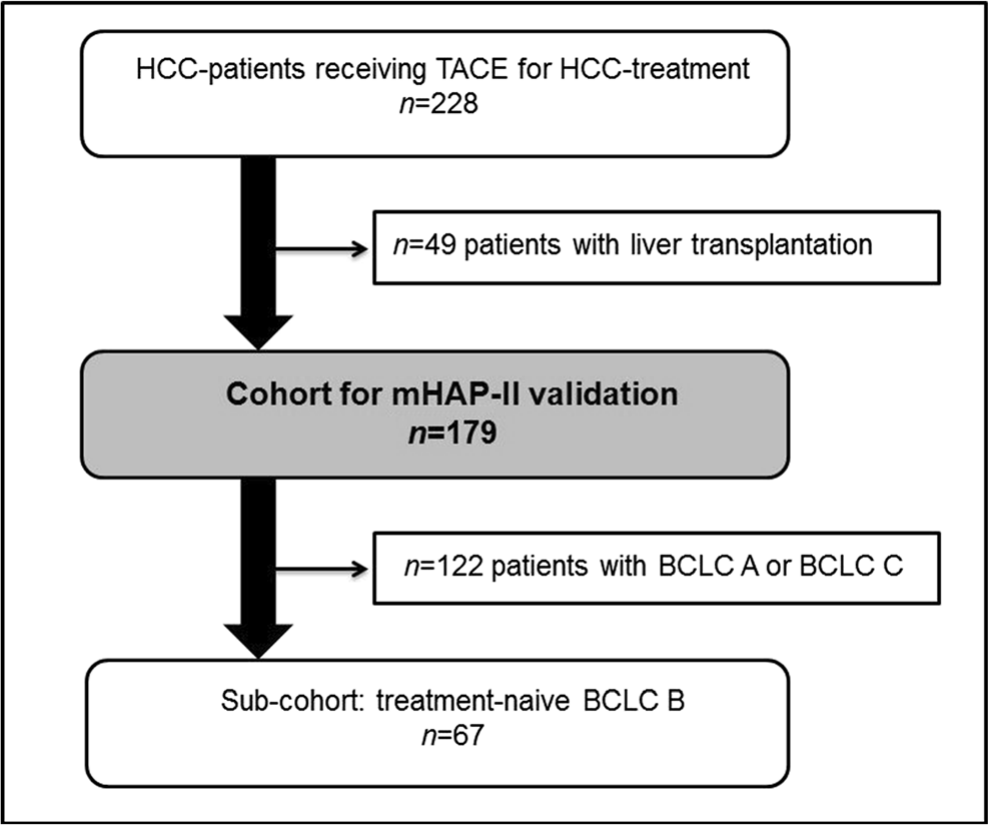
Patient selection

Patient selection and definition of subgroups is shown in Fig. 1. The cohort for survival analysis and validation of prognostic scores consisted of 179 patients, excluding 49 patients who received liver transplantation during follow-up. In a further analysis, treatment-naïve BCLC-B patients (*n* = 67) were analysed with regard to differentiating the discriminatory power of the HAP score and mHAP-II score. The decision to analyse this subgroup separately was based on the fact that current treatment guidelines only recommend TACE as the definitive treatment for patients with BCLC stage B, apart from those patients receiving TACE as a bridge to liver transplantation [20].

### TACE procedure

The arterial system was accessed through the common right femoral artery. After arterial puncture, a 4-F sheath (Terumo) and a 4-F straight catheter (Terumo) were introduced. An aortography was performed to assess the number and origin of hepatic arteries, and for the detection of abnormal anatomic blood supply to the liver in patients receiving their first treatment, especially if the anatomic blood supply was not clear from cross-sectional imaging. A 4-F Cobra (C2) or Sidewinder (SIM1) configured catheter (Cordis) was then introduced into the coeliac trunk and coeliacography was performed. From 2014 on, patients received a pre- and post-interventional cone beam CT. For selective catheterisation of hepatic arteries, a 2.7-F coaxial microcatheter was used (Progreat, Terumo). Selective (18%), or when possible super selective (82%), chemoembolisation was then performed using DC Bead particles (100–300 μm, BTG/Boston Scientific) loaded with 25–100 mg epirubicin. Drug-eluting microspheres were injected slowly under fluoroscopic control until near stasis was reached. After a time interval of approximately 10 min, selective control angiography was performed [21]. Follow-up CTs/MRIs to check for treatment response were performed every 3 months. The DEB-TACE procedure was repeated for patients with residual or recurrent tumours when feasible and necessary.

### Statistical analysis

Discrete/continuous data are reported as median/mean and interquartile range (IQR)/standard deviation, as appropriate. Categorical data are reported as counts and percentages. *T* tests and ANOVAs were used to compare the baseline characteristics of the groups, as appropriate. Survival time was measured with Kaplan-Meier curves from the day of the first DEB-TACE session until death or the last follow-up day. The baseline cumulative hazard after DEB-TACE was assessed by Breslow estimator and log-rank tests. Mann-Whitney *U* tests were used to pre-test the different scoring systems regarding their ability to differentiate between survivors and non-survivors. The discriminatory power of the different scores was assessed with ROC curves and Akaike information criterion (AIC). A Cox proportional hazard regression analysis was performed to assess the effect of the mHAP-II score variables in the study population. A difference noted with a *p* value of less than 0.05 was considered significant. Statistical analysis was performed with SPSS 25.0 (IBM SPSS).

### Ethics

This study protocol was performed according to the Declaration of Helsinki (1975) ethics guidelines and was approved by the local ethics committee. Due to the retrospective study design, written informed consent was waived.

## Results

### Baseline characteristics

**Fig. 2:**
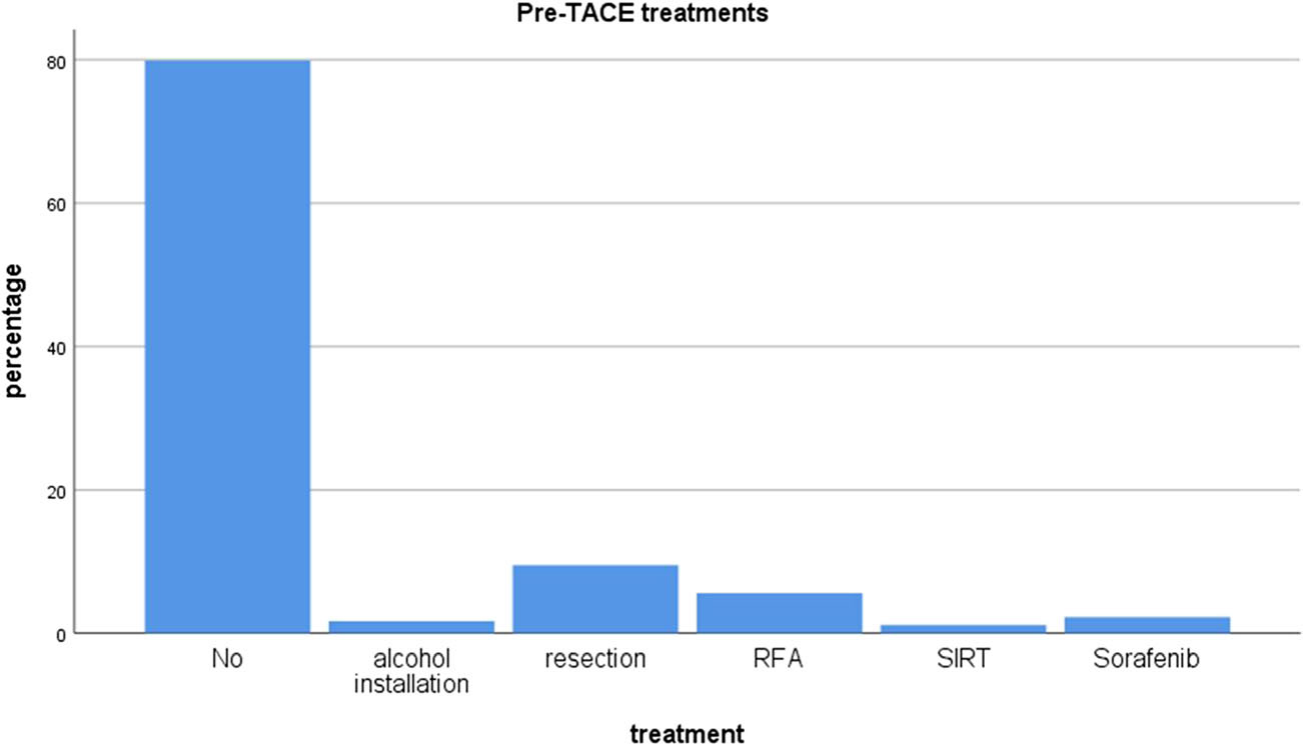
Pre-TACE treatments

One hundred seventy-nine patients were included in the scoring systems validation. Baseline characteristics, including treatments before TACE, are reported in Table 1 and Fig. 2. All HCC lesions were de novo lesions and for the most part did not receive any pre-treatment before TACE.

**Table 1:** Baseline characteristics

	Incl. LTX		Excl. LTX	
	*n*	Percentage	*n*	Percentage
Total patients	228	100%	179	100%
Sex				
Male	197	86%	155	87%
Female	31	14%	24	13%
Mean age (years)	75 (± 9)		77 (± 9)	
Aetiology				
HBV	25	11%	17	10%
HCV	68	30%	50	28%
Alcohol abuse	73	32%	55	31%
NASH	18	8%	15	8%
Other/not specified	44	19%	42	23%
Ascites	36	16%	23	13%
	Mean (SD)	Median (IQR)	Mean (SD)	Median (IQR)
Tumour size (mm)	44 (26)	39 (28–53)	47 (27)	42 (30–58)
Tumour number (*n*)	3.3 (9.3)	2.0 (1.0–3.0)	3.2 (7.6)	2.0 (1.0–3.0)
Alpha-fetoprotein (μg/l)	310.3 (1027.9)	14.0 (6.0–97.0)	386.2 (1152.6)	17.0 (6.3–165.5)
Serum albumin (g/dl)	3.7 (0.6)	3.9 (3.3–4.2)	3.8 (0.5)	3.9 (3.4–4.2)
Total bilirubin (mg/dl)	1.6 (3.9)	1.0 (0.9–1.0)	1.3 (0.9)	1.0 (0.9–1.0)
TACE (*n*)	2.6 (1.5)	2.0 (1.5–3.0)	2.8 (1.6)	3.0 (2.0–3.0)
Overall survival (months)	55.1 (3.6)	36.6 (29.6–43.6)	35.8 (2.5)	29.4 (24.4–34.5)
	*n*	Percentage	*n*	Percentage
1-year survival rate		84%		80%
2-year survival rate		66%		58%
3-year survival rate		53%		41%
4-year survival rate		40%		25%
5-year survival rate		34%		16%
Child-Pugh				
A	161	71%	136	76%
B	63	28%	41	23%
C	4	2%	2	1%
BCLC				
0	4	2%	2	1%
A	67	29%	47	26%
B	108	47%	88	49%
C	44	19%	38	21%
D	5	2%	4	2%
HAP				
A	16	7%	15	8%
B	93	41%	73	41%
C	97	43%	71	40%
D	22	10%	20	11%
mHAP-II				
A	12	5%	11	1%
B	66	29%	53	30%
C	101	44%	73	41%
D	49	21%	42	23%

*BCLC*, Barcelona Clinic Liver Cancer staging system; *HAP*, hepatoma arterial prognostic score; *HBV*, hepatitis B virus; *HCV*, hepatitis C virus; *IQR*, interquartile range; *LTX*, liver transplantation; *mHAP-II*, modified hepatoma arterial prognostic score; *n*, number; *NASH*, non-alcoholic steatohepatitis; *SD*, standard deviation; *TACE*, trans-arterial chemoembolisation

Eighty-seven patients underwent additional treatments subsequent to their treatment with TACE: Systemic chemotherapy (*n* = 30), radiofrequency ablation (RFA) (*n* = 27), selective internal radio therapy (SIRT) (*n* = 16), resection (*n* = 8) and other (*n* = 6).

### Survival and follow-up

The median survival of the validation cohort of 179 patients was 29.4 months (IQR 24.4–34.5). One-, 2-, 3-, 4- and 5-year survival rates were 80%, 58%, 41%, 25% and 16%, respectively. The median follow-up time was 42.0 months (95% CI 38.9–53.7). A total of 126 patients died by the end of the follow-up period (January 2019) and 40 patients were lost to follow-up.

### Discrimination power of mHAP-II score, HAP score and BCLC

In Mann-Whitney *U* tests, BCLC (*U* = 2404.5, *p* = 0.01) and mHAP-II score (*U* = 2579.5, *p* = 0.03) could differentiate between survivors and non-survivors. CLIP, HAP, MESH and OKUDA scores failed to differentiate between survivors and non-survivors (each *p* > 0.05). As the HAP score only narrowly missed statistical significance (*U* = 2680.5, *p* = 0.06), it was also included in further analysis.

**Fig. 3:**
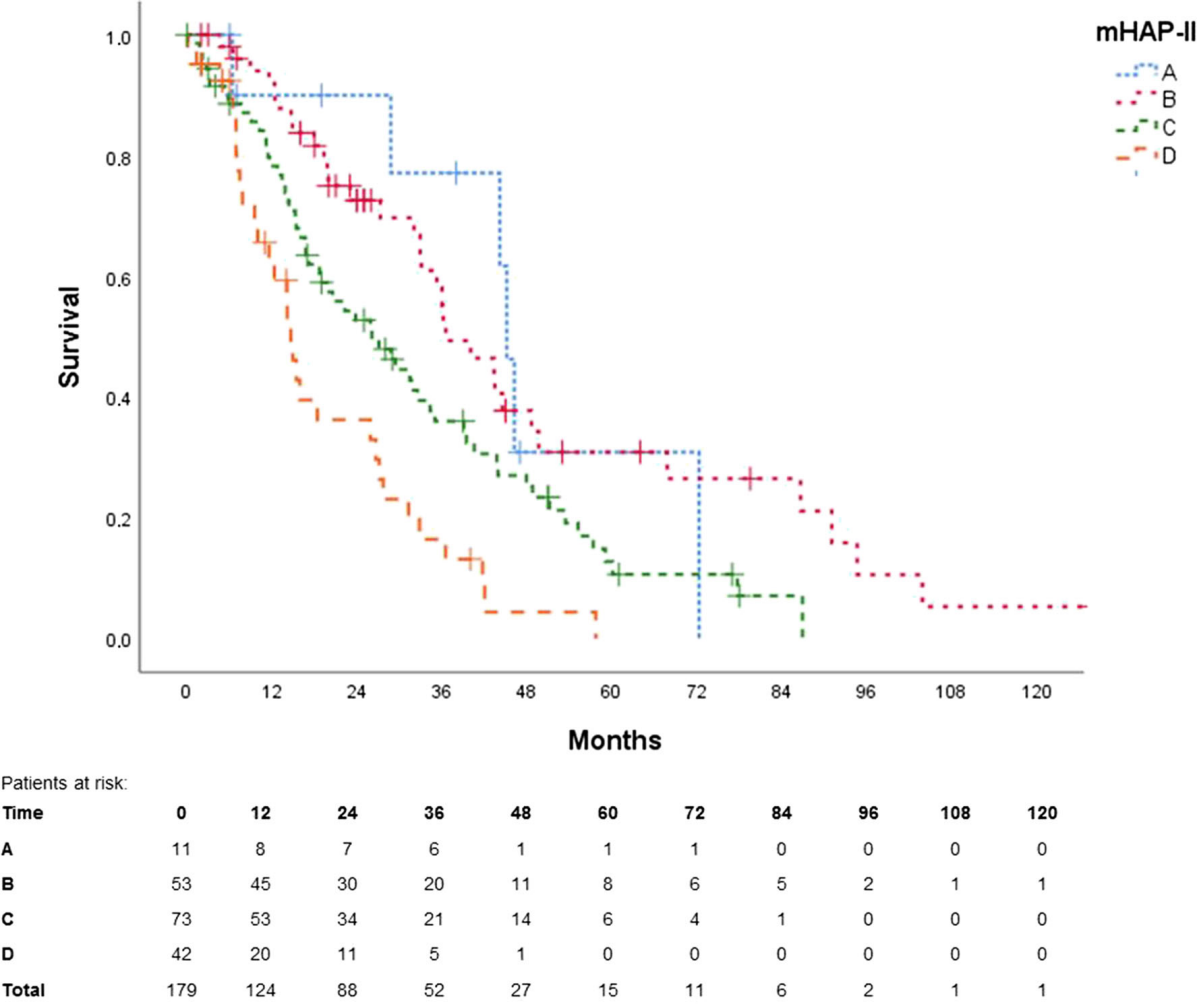
Kaplan-Meier curve for mHAP-II score

**Fig. 4:**
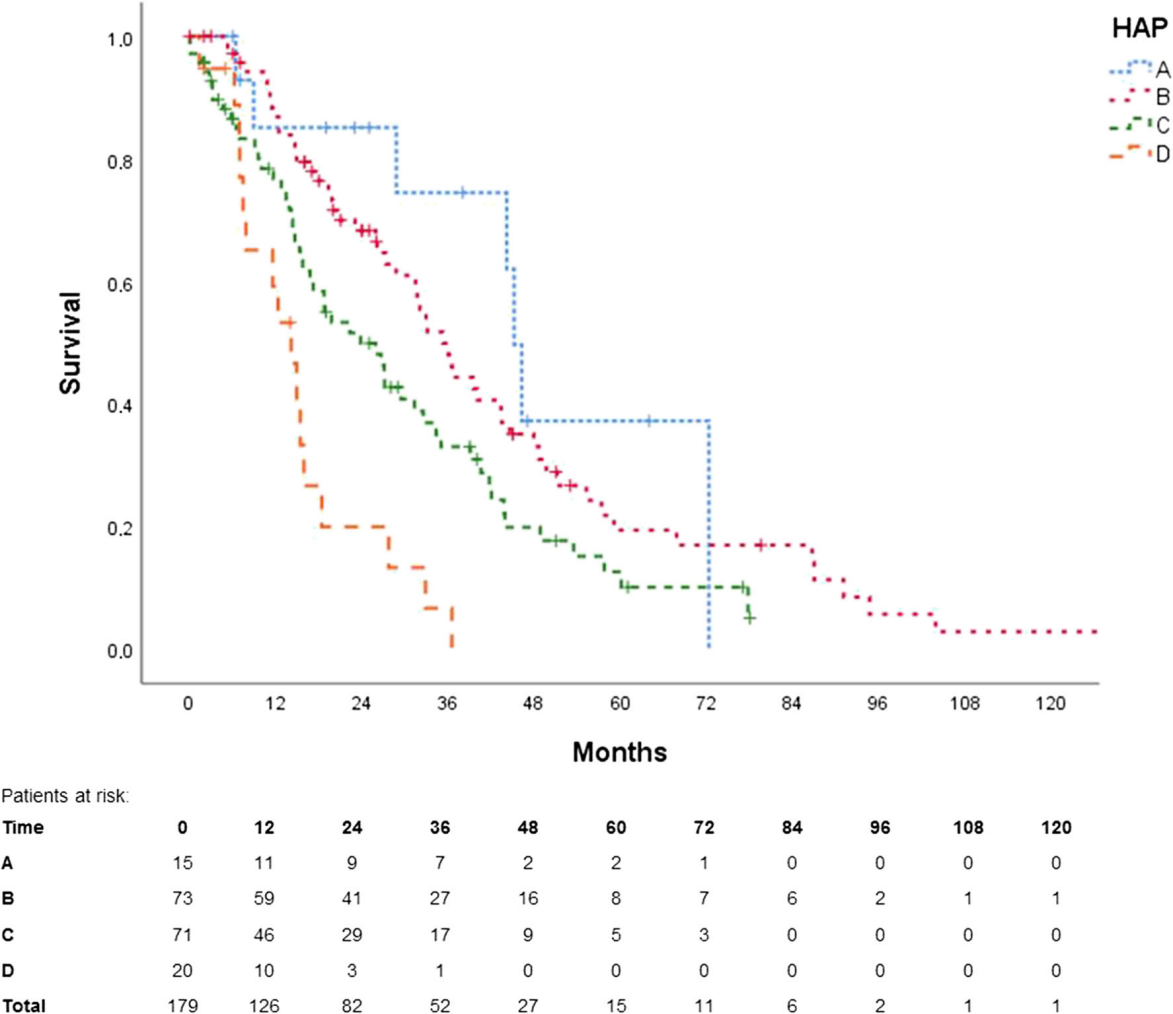
Kaplan-Meier curve for HAP score

**Fig. 5:**
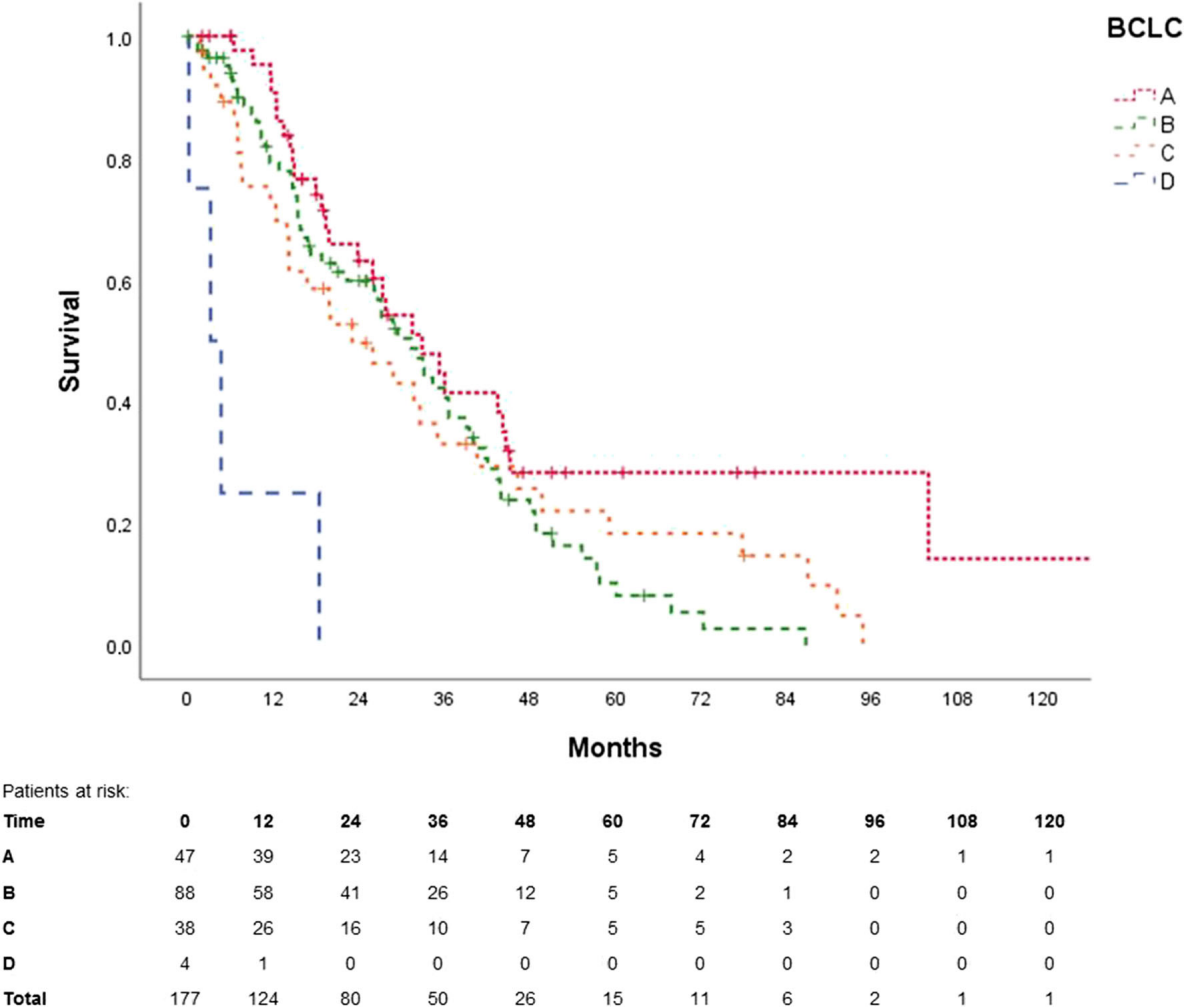
ROC curve for HAP score, mHAP-II score and BCLC for the sample (*n* = 179) excluding LTX

**Fig. 6:**
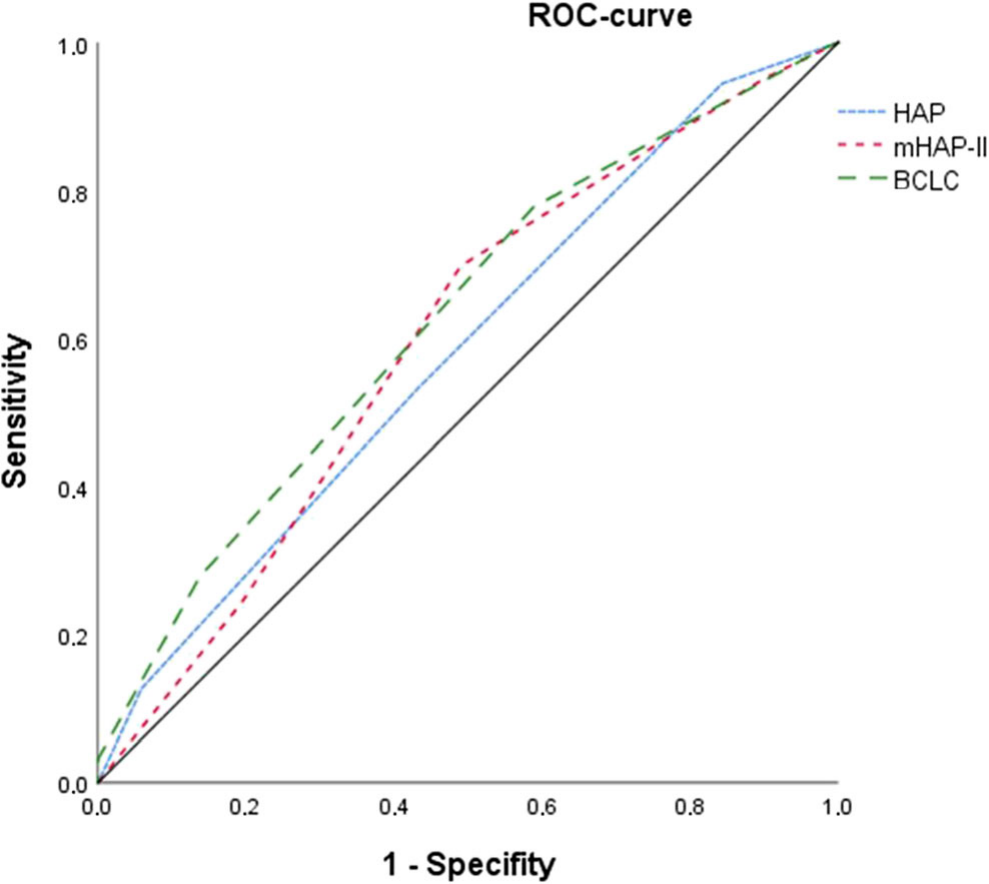
ROC curves for HAP score and mHAP-II score for treatment naive BCLC-B patients

Figures 3, 4, 5 and 6 show the Kaplan-Meier curves and ROC curves for mHAP-II score, HAP score and BCLC stage respectively. Median survivals and results of the ROC curves are reported in Table 2.

**Table 2:** Discrimination ability for HAP score, mHAP-II score and BCLC for patients undergoing TACE, excluding patients with subsequent liver transplantation

	Survival after TACE in months				AUC-ROC
	Median	95% CI		*p* values	
mHAP-II					0.60
A	45.2	42.7	47.7	A vs. B: *p* = 0.86; A vs. C: *p* = 0.21; A vs. D: *p =* 0.001	
B	36.6	26.6	46.6	B vs. C: *p* = 0.01; B vs. D: *p* < 0.001	
C	26.1	16.6	35.6	C vs. D: *p* = 0.004	
D	14.7	13.0	16.4		
HAP					0.58
A	45.2	42.4	47.9	A vs. B: *p* = 0.39; A vs. C: *p* = 0.04; A vs. D: *p* < 0.001	
B	35.3	30.1	40.4	B vs. C: *p* = 0.02; B vs. D: *p* < 0.001	
C	23.8	13.9	33.8	C vs. D: *p* = 0.01	
D	14.1	9.9	18.3		
BCLC					0.63
A	32.8	22.5	43.2	A vs. B: *p* = 0.05; A vs. C: *p* = 0.08; A vs. D: *p* < 0.001	
B	31.3	25.0	37.6	B vs. C: *p* = 0.59; B vs. D: *p* < 0.001	
C	23.0	11.3	34.8	C vs. D: *p* = 0.001	
D	3.2	0	7.6		

*AUC-ROC*, area under the curve for receiver operating characteristics; *BCLC*, Barcelona clinical liver classification; *HAP*, hepatoma arterial embolisation score; *CI*, confidence interval; *mHAP-II*, modified hepatoma arterial embolisation prognostic score; *TACE*, trans-arterial chemoembolisation

Except for group A vs. B and group A vs. C, the mHAP-II score could successfully differentiate between its different subgroups (each *p* < 0.05) with an AUC of 0.60 in ROC (95% CI 0.50–0.70). AUC in ROC for 1-, 2- and 3-year survival rates was 0.41 (95% CI 0.32–0.50), 0.38 (95% CI 0.30–0.47) and 0.32 (95% CI 0.22–0.43), respectively.

The HAP score performed similarly, failing only to discriminate group A from group B. AUC in ROC was 0.58 (95% CI 0.49–0.68).

The BCLC system failed to differentiate between groups A vs. B, A vs. C and B vs. C. AUC in ROC was 0.63 (95% CI 0.54–0.72).

The AUC in ROC from HAP, mHAP-II and BCLC did not differ significantly from each other (HAP vs. mHAP-II, *p* = 0.28; HAP vs. BCLC, *p* = 0.32; mHAP-II vs. BCLC, *p* = 0.55).

The AIC for mHAP-II score was 21.8 (*p* = 0.03), whilst it was 20.6 (*p* = 0.04) for HAP score and 18.92 (*p* = 0.01) for BCLC respectively.

### Analysis of the subcohort of treatment-naïve BCLC-B patients

Treatment-naïve patients with BCLC-B had an overall median survival of 26.7 months (95% CI 12.5–40.8). Median survivals and AUC-ROC results for HAP score and mHAP-II score are reported in Table 3.

**Table 3:** Discrimination ability for HAP score, mHAP-II score for treatment-naïve BCLC stage B patients

	Survival after TACE in months				AUC-ROC
	Median	95% CI			
mHAP-II				*p* values	0.51
A	n.a.	n.a.	n.a.		
B	40.1	33.7	46.4	B vs. C: *p* = 0.54; B vs. D: *p* = 0.01	
C	22.3	11.3	33.3	C vs. D: *p* = 0.05	
D	14.9	6.4	23.5		
HAP					0.52
A	8.9	n.a.	n.a.	A vs. B: *p* = 0.13; A vs. C: *p* = 0.33; A vs. D: *p* < 0.87	
B	36.6	17.1	56.0	B vs. C: *p* = 0.16; B vs. D: *p* = 0.03	
C	15.4	8.6	22.3	C vs. D: *p* = 0.36	
D	15.4	0	35.2		

*AUC-ROC*, area under the curve for receiver operating characteristics; *BCLC*, Barcelona clinical liver classification; *CI*, confidence interval; *HAP*, hepatoma arterial embolisation score; *mHAP-II*, modified hepatoma arterial embolisation prognostic score; *TACE*, trans-arterial chemoembolisation

The mHAP-II score was able to differentiate between group B vs. D and C vs. D with an AUC of 0.51 (95% CI 0.31–0.72). HAP score discriminated group B vs. D with an AUC of 0.52 (95% CI 0.33–0.71). AUC for HAP and mHAP-II did not differ significantly from each other.

AIC was 18.72 (*p* = 0.31) for mHAP-II score and 16.01 (*p* = 0.22) for HAP score.

### Identification of independent prognostic factors of mHAP-II score

Cox regression analysis of the cohort (excluding patients with subsequent LTX (*n* = 179 patients)) was performed, and parameters of the mHAP-II score were evaluated. Cox regression analyses revealed that a tumour number equal to or greater than two (HR 1.54), AFP > 400 μg/l (HR 1.14), serum albumin < 3.6 g/dl (HR 1.63) and total bilirubin > 0.9 mg/dl (HR 1.58) were independent risk factors in our population (Table. 4). The parameter “tumour size > 70 mm” failed significance (*p* = 0.14).

**Table 4:** Association between death and selected variables

Variable	HR	95% CI		*p*
Tumour number ≥ 2	1.54	1.05	2.26	0.03
Tumour size > 70 mm	1.46	0.89	2.40	0.14
AFP > 400 μg/l	1.72	1.14	2.59	0.01
Serum albumin < 3.6 g/dl	2.51	1.63	3.86	< 0.01
Bilirubin > 0.9 mg/dl	1.58	1.02	2.44	0.04

*AFP*, alpha-fetoprotein; *CI*, confidence interval; *HR*, hazard ratio

## Discussion

Current guidelines recommend TACE for HCC patients with BCLC stage B [20]. However, this group of patients is very heterogeneous with regard to tumour aetiology, liver function status and clinical presentation [22]. The expected benefit from TACE therefore varies significantly within this group, making prognosis and ultimate treatment decisions difficult.

Several research groups addressed this issue by developing different scoring systems to further subdivide patients eligible for TACE. Commonly used scores include the HAP score [9] and its later-developed successor, the mHAP-II score [11].

Although the mHAP-II score showed improved performance compared with HAP score and mHAP score in the initial paper by Park et al [11], the question remains as to whether the results are transferrable to a real-life western population. Furthermore, evolving new techniques, such as DEB-TACE, were not addressed in the original publication.

The analysed cohort represented a typical northern European HCC cohort, in which alcohol and HCV are the most important risk factors compared with south Asia and Africa, where HBV infection is more predominant (64% vs. 13% in a western population) [23]. This is an important difference compared with the original publication by Park et al, as there are known differences in tumour biology depending on the underlying origin of liver cirrhosis [24].

The direct applicability of the HAP and mHAP scores has not yet been determined. The focus of this paper was to investigate and compare different scoring systems in a western HCC cohort treated exclusively with DEB-TACE. Firstly, we were interested in determining which of the common scoring and staging systems (HAP, mHAP-II, CLIP, OKUDA, MESH and BCLC) would be of interest for a further comparison. In Mann-Whitney *U* tests, only BCLC and mHAP-II score showed significant results in terms of survival prediction. As the HAP score only narrowly missed statistical significance, it also was included in further analyses with Kaplan-Meier curves, ROC and AIC.

The median overall survival of our cohort was shorter than in the study by Parks et al [11] (29.4 vs. 40.5 months) and from other data reported by Burrel et al from a Spanish population of 104 patients treated with DEB-TACE (48.6 months) [25]. This difference is most likely due to the fact that Burrel’s cohort presented with a better preserved liver function (95% Child-Pugh A and 40% BCLC A vs. 76% Child-Pugh A and 26% BCLC A in our cohort) [25]. However, the survival in our cohort was longer compared with other data from comparable cohorts. Cappelli et al worked with a population that showed a median survival of 24.6 months [26], and reported more cases with anti-HCV positivity in their cohort (61.5% vs. 28%) and less cases with concomitant alcohol abuse (10.2% vs. 31%). Puchol et al reported a median survival of 22.4 months in a Spanish cohort of 47 patients treated with adriamycin-loaded beads, and also reported a much higher proportion of HCV infection (72.2% vs. 28%) and a lower rate of alcoholism (12.5% vs. 31%) compared with our study population [27]. Again, differences in overall survival are most likely due to different compositions of the investigated cohorts and slightly different TACE procedures.

Kaplan-Meier curves were able to discriminate between almost every mHAP-II group; however, difficulty arose when differentiating between group A vs. B and group A vs. C. This might be because our population had a rather small mHAP-II group A (*n* = 11), compared with B (*n* = 53), C (*n* = 73) and D (*n* = 42).

One-, 2-, 3-, 4- and 5-year survival rates (80%, 58%, 41%, 25% and 16%) were comparable with Park et al (81%, 66%, 52%, 40% and 33%) and were higher than those reported by Chen et al (1 year 60%, 2 years 49%, 5 years 8%) and Cappelli et al (1 year 76%; 3 years 34%) [11, 26, 28]. Reasons for the reported differences are most likely to be found in the different compositions of the analysed populations. Chen et al, for example, worked with a population that had a much higher proportion of patients in Child-Pugh class B (30.5% vs. 23%) and BCLC-B (91.8% vs. 49%) compared with our population, which might explain the lower 1-, 2-, and 5-year survival rates compared with our study.

Further investigation of the quality of mHAP-II score in terms of predicting overall survival showed only moderate predictive power with an AUC of 0.60 (95% CI 0.50–0.70), representing poor accuracy. Cappelli et al also published similar results (AUC 0.61; 95% CI 0.57–0.65) with regard to overall survival [26].

Regarding the AUC of mHAP-II score focusing on 1-, 2-, and 3-year survival rates, the score failed to accurately predict survival (AUC 0.41, 0.32 and 0.32 respectively). Whilst Park et al reported an AUC for mHAP-II of 0.72 for 3-year survival and 0.73 for 5-year survival [11], the mHAP-II score in our population failed in terms of predictability of 1-, 2- and 3-year survival.

AIC was 21.8 (*p* = 0.03), indicating a sufficient but only slightly increased performance of the mHAP-II score in our population, compared with 20.6 (*p* = 0.04) for HAP score and 18.9 (*p* = 0.01) for BCLC, respectively. Other authors report much higher values for the AIC for mHAP-II score and HAP score (2544 for mHAP-II score and 2554 and 1361 for HAP score respectively); however, comparisons between different populations should be exercised only with caution, as AIC only allows for comparisons within the same sample [26, 28, 29].

Cox regression revealed that a tumour number equal or greater to two (HR 1.54), AFP > 400 μg/l (HR 1.14), serum albumin < 3.6 g/dl (HR 1.63) and total bilirubin > 0.9 mg/dl (HR 1.58) were independent risk factors in our population, excluding patients with subsequent LTX. The parameter “tumour size > 70 mm”, derived from the original publication by Park et al, failed significance (*p* = 0.14). This was a very interesting finding, as it indicates that tumour size alone might not be a predictive factor for the outcome in DEB-TACE compared with cTACE. This indicates that DEB-TACE might be of advantage in large tumours compared with cTACE.

Since current guidelines suggest that only patients with BCLC stage B should be treated with TACE as definitive treatment [20], treatment-naïve patients with BCLC stage B HCC were analysed as a subcohort (*n* = 67). Statistical analysis showed that the mHAP-II score was only able to successfully differentiate between group B vs. D and C vs. D. The AUC was 0.51, indicating that the mHAP-II score failed to reliably predict overall survival in this subcohort.

DEB-TACE and cTACE have been compared in several studies. However, it is still unclear whether the outcomes significantly differ from each other. In terms of overall survival, several studies showed similar outcomes, whilst others showed a significant benefit from DEB-TACE [30–32]. The same applies to relevant side effects [19, 33, 34]. As the aforementioned predictive scoring systems were only validated in cTACE cohorts, it was of special interest to investigate this issue in a DEB-TACE-only cohort. The advantage of using this cohort is the ability to rule out the use of mixed TACE techniques as an additional confounding factor. The question of whether the mHAP-II score works in mixed populations was already addressed by other authors, such as Kirstein et al [35]. They emphasised the impact of baseline tumour characteristics and patient-related factors on long-term survival, a finding that is supported by our study.

A possible confounding variable was the technical development of the TACE technique within the timespan of the study. The majority (81%) of patients were treated super selectively and 19% received a selective embolisation. However, the basic technical approach remained highly standardised throughout the entire timespan of the study and therefore the possibility of a significant alteration in technique which could affect study outcomes is unlikely.

In conclusion, our work shows that the mHAP-II score can predict survival outcomes of western HCC patients undergoing DEB-TACE and further subdivide the heterogeneous group of patients receiving TACE. However, because the study is underpowered, true survival prediction may be more difficult to infer. In our assessment, future analysis trends would benefit from the use of continuous rather than categorical variables [36]. However, the strength of our study lies in its advances in predicting benefit for western BCLC stage B HCC patients receiving DEB-TACE, which brings us one step closer to creating a superior prediction model for this particular patient population.

## Abbreviations

AFP: Alpha-fetoprotein
AIC: Akaike information criterion
ANOVA: Analysis of variance
AUC: Area under the curve
BCLC: Barcelona Clinic Liver Cancer staging system
CI: Confidence interval
CT: Computer tomography
DEB: Drug-eluting bead
EASL: European Association for the Study of the Liver
ECOG: Eastern Cooperative Oncology Group
HAP: Hepatoma arterial prognostic score
HBV: Hepatitis B virus
HCV: Hepatitis C virus
HR: Hazard ratio
IQR: Interquartile range
LTX: Liver transplantation;
mHAP-II: Modified hepatoma arterial prognostic II score
mRECIST: Modified response evaluation criteria in solid tumours
MRI: Magnet resonance imaging
NASH: Non-alcoholic steatohepatitis
PS: Performance status
RFA: Radiofrequency ablation
ROC: Receiver operating characteristics
SD: Standard deviation
SIRT: Selective internal radio therapy
TACE: Trans-arterial chemoembolisation
